# Non-radiative relaxation of photoexcited chlorophylls: theoretical and experimental study

**DOI:** 10.1038/srep13625

**Published:** 2015-09-08

**Authors:** William P. Bricker, Prathamesh M. Shenai, Avishek Ghosh, Zhengtang Liu, Miriam Grace M. Enriquez, Petar H. Lambrev, Howe-Siang Tan, Cynthia S. Lo, Sergei Tretiak, Sebastian Fernandez-Alberti, Yang Zhao

**Affiliations:** 1 Department of Energy, Environmental and Chemical Engineering, Washington University, Saint Louis, Missouri 63130, USA; 2 Division of Materials Science, Nanyang Technological University, Singapore 639798; 3 Division of Chemistry and Biological Chemistry, School of Physical and Mathematical Sciences, Nanyang Technological University, Singapore 637371; 4 Biological Research Center, Hungarian Academy of Sciences, 6726 Szeged, Temesvari krt. 62, Hungary; 5 Theoretical Division, Center for Nonlinear Studies (CNLS), and Center for Integrated Nanotechnologies (CINT), Los Alamos National Laboratory, Los Alamos, New Mexico 87545, USA; 6 Universidad Nacional de Quilmes, Roque Saenz Pea 352, B1876BXD Bernal, Argentina

## Abstract

Nonradiative relaxation of high-energy excited states to the lowest excited state in chlorophylls marks the first step in the process of photosynthesis. We perform ultrafast transient absorption spectroscopy measurements, that reveal this internal conversion dynamics to be slightly slower in *chlorophyll B* than in *chlorophyll A*. Modeling this process with non-adiabatic excited state molecular dynamics simulations uncovers a critical role played by the different side groups in the two molecules in governing the intramolecular redistribution of excited state wavefunction, leading, in turn, to different time-scales. Even given smaller electron-vibrational couplings compared to common organic conjugated chromophores, these molecules are able to efficiently dissipate about 1 eV of electronic energy into heat on the timescale of around 200 fs. This is achieved via selective participation of specific atomic groups and complex global migration of the wavefunction from the outer to inner ring, which may have important implications for biological light-harvesting function.

The process of light-harvesting that marks the beginning of photosynthesis in different photosynthetic organisms is carried out by a variety of pigment molecules including chlorophylls, bacteriochlorophylls and carotenoids^1^. On the one hand, as a part of antenna complexes, chlorophylls absorb photons and subsequently transfer the excitation energy to neighboring pigments, while on the other hand, they even participate in charge separation and electron transfer inside the reaction center complexes^2^. The dynamics of the photoexcited states in various natural light-harvesting systems has thus attracted a huge amount of research efforts^2^,^3^,^4^,^5^,^6^,^7^,^8^,^9^. In general, the inter-molecular energy transfer in chlorophylls and bacteriochlorophylls, which is generally thought to be associated with the lowest excited state, called the *Q*_*y*_ excitation, is the typical focus of such studies owing to the ultra-high efficiency with which the excitation energy reaches the reaction center complexes on a sub-100 ps timescale to trigger the chemical energy conversion process^4^. However, the preceding step of ultra-fast (sub-ps) relaxation of the higher energy excited states to the lowest excited state has not received thorough attention so far. Such intra-molecular excited state dynamics is in fact crucially relevant to certain aspects of this vastly complicated process. Within the widely used four-orbital model for porphyrins proposed by Gouterman^10^,^11^, the absorption spectra of chlorophylls are composed of four bands, namely, the low energy *Q*_*y*_ and *Q*_*x*_ bands, and the high energy *B*_*x*_ and *B*_*y*_ bands. All the chlorophylls exhibit strong absorption in the *B* band (~400–470 nm)^2^, which implies a substantial likelihood for a chlorophyll molecule to get photoexcited at a high-energy state in the *B* band. In chlorophyll *a*, the *Q*_*x*_ and *Q*_*y*_ bands have been shown to be mixed due to strong vibronic coupling, implying that the Gouterman model of purely independent transitions is not completely accurate for chlorophylls^12^. Under ambient light conditions, a relatively rapid decay to the lowest excited state is thus essential to initiate the inter-molecular excitation energy transfer that takes place via the incoherent Förster resonance energy transfer mechanism^13^,^14^ or through quantum coherence in some photosynthetic complexes^4^,^15^. On the other hand, a regulatory mechanism known as non-photochemical quenching (NPQ) is activated to protect the delicate photosynthetic machinery from the excess excitation energy generated upon relatively intense incident light^15^,^16^. NPQ essentially involves the deactivation of excited states as heat along a safe channel to the ground state, so that dangerous triplet excited states are not formed through inter-system crossing. It is thus important to study the behavior of high-energy excited states by elucidating underlying mechanisms of internal conversion processes in biological systems.

The internal conversion timescales in chlorophyll *a* (ChlA) in a number of solvents were first probed experimentally by Shi *et al.* with the use of a time-resolved fluorescence depletion spectroscopy technique^17^. The time constants in different aprotic solvents for the *B* → *Q*_*y*_ decay were found to be in a range from 146 ± 10 fs to 260 ± 10 fs whereas those for *Q*_*x*_ → *Q*_*y*_ ranged from 100 ± 10 fs to 226 ± 10 fs. The internal conversion time was found to increase with an increase in the dielectric constant of the solvent. This was followed by the studies of fluorescence depletion spectra by employing the density matrix theory and the transient linear susceptibility theory where internal conversion timescales in agreement with the experimental results were obtained^18^,^19^. The internal conversion processes have also been extensively investigated in bacteriochlorophyll *a* molecules with the naturally occurring central coordinated metal ion Mg^2+^ 20, as well as their Zn^2+^ and Ni^2+^ ion analogues^21^,^22^. Reimers *et al.* estimated the *Q*_*x*_ → *Q*_*y*_ decay constant based on a vibronic coupling model from 99–134 fs depending on the solvent. The location of the *Q*_*x*_ band in ChlA is of particular interest, with two separate x-polarized bands occurring in spectra; the “traditional” assignment of *Q*_*x*_ is the higher-energy x-polarized component, whereas the “modern” assignment is the lower-energy component. The role of strong vibronic coupling and mixing between *Q*_*x*_ and *Q*_*y*_ bands in ChlA is thought to significantly influence internal conversion properties^12^. In secondary photosynthetic pigments such as carotenoids, significant efforts have revealed many interesting features of the internal conversion processes, which are perceived to be important in understanding their dual role in transferring the excitation energy absorbed in the blue region to the chlorophylls, and in participating in the photoprotective mechanisms of NPQ^23^,^24^,^25^,^26^,^27^,^28^. Recent studies on the conversion from the optically allowed *S*_2_ state to the forbidden *S*_1_ state in *β*-carotene propose a single-step mechanism^23^,^24^, in contrast to the earlier conjecture of a participating intermediate state^25^. Presence of such an intermediate state was found to be important in explaining the dependence of internal conversion rate on the conjugation length^26^. Apart from the few studies on the photoactive pigments, nonradiative relaxation processes in a number of other molecules or compounds^29^,^30^,^31^,^32^, including biological macromolecules such as DNA^33^ and RNA^34^, have also been extensively investigated typically with the use of ultrafast femtosecond spectroscopic techniques. Furthermore, a recent computational study employing density functional theory based calculations on chlorophyll-carotenoid aggregates has proposed possible involvement of vibrational relaxation of carotenoid excited states in enhancing *B* to *Q*-band internal conversion in chlorophyll molecules^35^. As described later in the text, in this work we have studied the internal conversion in monomeric chlorophyll molecules, the computational approach used can be readily extended in future to probe features of NPQ by studying systems such as homodimers of chlorophylls or heterodimers of chlorophylls and carotenoids, which are speculated to act as excitation quenching sites in natural light-harvesting complexes.

In this work, we focus on studying theoretically and experimentally, the intramolecular relaxation of photoexcited states of two important chlorophyll molecules - ChlA and chlorophyll *b* (ChlB), shown in Fig. 1. ChlA is the most abundant natural photosynthetic pigment that is found in every green plant, followed in abundance by ChlB^1^. Structurally, these two chlorophyll pigments differ only by an extra carbonyl oxygen at the *R*_7_ position (ChlB), yet are known to exhibit marked differences in their absorption spectra. We have systematically investigated the *B* to *Q*-band internal conversion processes in these two molecules by employing Non-Adiabatic Excited State Molecular Dynamics (NA-ESMD) simulations. This methodology has been successfully used to simulate the ultrafast intramolecular redistribution and relaxation of the excess of electronic energy after photoexcitation in many large organic conjugated chromophores^36^,^37^,^38^. The role played by relatively minor structural differences between these molecules in the dynamics of relaxation of high-energy excited states has been revealed via analysis based on evolution of transition density localization.

## Results and Discussion

### Transient Absorption Spectroscopy

The dynamics of internal conversion in ChlA and ChlB solutions after excitation in the high energy *B* band was probed by femtosecond transient absorption (TA) spectroscopy (full details are given in Experimental). While both pigments are pumped at 442 nm, the probe wavelength are set to the respective *Q*_*y*_ region. The TA spectra in Fig. 2(a) are characterized by a negative band with a maximum at 670 nm for ChlA and 650 nm for ChlB, due to ground-state bleaching and stimulated emission, and a positive signal below 650 nm due to excited-state absorption. From the kinetic traces of the negative TA in a 1 ps time window in Fig. 2(b), it is evident that the dynamics in both ChlA and ChlB is very similar. Both curves display an initial rapid increase of the signal followed by a second, slower phase. The initial rise depicts the appearance of ground-state bleaching following excitation in the *B* band by the 55 fs wide pump pulse. After the initial excitation, the slower rise is presumably due to stimulated emission appearing along with the population of the emitting lowest excited state (*Q*_*y*_). Thus, the slower rise of the TA signal reflects the dynamics of IC from the *B* band to *Q*_*y*_.

To obtain a quantitative measure of the time constant of internal conversion, the TA data were subjected to global multiexponential analysis^39^, using the program Glotaran^40^, whereby the TA kinetics at all wavelengths was described by a single-exponential rise convoluted with a Gaussian (55 fs FWHM) instrument response function. More precisely, a two-component sequential kinetic scheme was used - *S*_*n*_ → *S*_1_ - with one component representing higher *S*_*n*_ excited states and a quasi-stationary component (with a fixed lifetime of 5 ns), representing the *S*_1_ state. The lifetime of the *S*_*n*_ states and the pump-probe spectra of the *S*_*n*_ and *S*_1_ states (species-associated absorption difference spectra, SADS) were obtained by fitting the model to the experimental TA data. The model also accounted for chirp (group velocity dispersion) and coherent artifact. The best fit was obtained with lifetimes of 143 fs for ChlA and 162 fs for ChlB (standard errors of fit ca. 5%). The SADS are shown in Fig. 3(a,b) for ChlA and ChlB, respectively. In both cases, the initial SADS has a smaller amplitude, blue-shifted maximum (664 nm and 647 nm for ChlA and ChlB, respectively), and no appreciable excited-state absorption at 600–640 nm, confirming that the spectrum represents higher excited states, whereas the second SADS represents the emitting *S*_1_ state. Owing to these spectral differences, global analysis of the TA could extract the time constant of *S*_*n*_ → *S*_1_ internal conversion with much greater accuracy than what would be possible by analyzing time traces at single probe wavelengths. From this analysis it can be concluded that internal conversion time constant in ChlB is very close to, or within the error estimates, slightly larger than that in ChlA.

### Ground State Molecular Dynamics and Absorption Spectra

To model internal conversion processes in ChlA and ChlB we employ the non-adiabatic excited-state molecular dynamics (NA-ESMD) simulations (full details are given in Computational Methods), which require sampling of the ground state potential energy surfaces (PESs). Generation of such an ensemble consisting of 500 input configurations is achieved via ground state molecular dynamics simulations (details are given in Ground State Molecular Dynamics) at 300 K. In order to guide the set-up of subsequent excited state dynamics, we calculated the absorption spectrum for each molecule after averaging over the profiles of excited state energies and the corresponding oscillator strengths for the 500 configurations. Figure 4 shows a comparison of the calculated absorption spectra of ChlA and ChlB to their corresponding experimentally measured steady-state spectra in ethanol. Although the calculated excited state energies and oscillator strengths cannot be compared precisely with experimental results owing to the well known shortcomings of the semi-empirical methods, several key features of the experimental spectra are still found to be well reproduced in the calculated spectra. We note that although semiempirical methods are poor predictors of absolute excitation energies, most experimental features are retained when comparing relative excitation energies between ChlA and ChlB as well as between excited states in each molecule. First, the *Q*_*y*_ and *Q*_*x*_ bands compare well with the experimental spectra in terms of energetic ordering, with ChlB states having the higher energies, followed by ChlA. Second, the relative oscillator strengths of the *Q*_*y*_ bands are in agreement to those in experiment, where ChlB has lower oscillator strength than ChlA. ChlB also has the most distinguishable *Q*_*x*_ band of the two chlorophyll molecules, but *Q*_*x*_ band energies and oscillator strengths in experimental spectra are more difficult to ascertain than the Soret and *Q*_*y*_ bands. Finally, the energy gap between the well distinguished Soret and *Q*_*y*_ band peaks in the experiment spectrum is 1.01 eV in ChlA and 0.76 eV in ChlB. The calculated spectrum, on the other hand, yields an energy gap of 1.24 eV in ChlA and 1.12 eV in ChlB, exhibiting a trend that is in reasonable agreement with the experimental counterpart. It is important to note that the calculated *Q*_*x*_ bands in both ChlA and ChlB are closer in excitation energy to their respective *B* bands than the *Q*_*y*_ bands, a trend which is not supported by experiment. Thus, this study may reproduce trends in overall *B* → *Q*_*y*_ internal conversion more accurately than in *B* → *Q*_*x*_ and *Q*_*x*_ → *Q*_*y*_ internal conversion. Notably, such reproduction in the trends of experimental gaps between excited states bears significance for the NA-ESMD results and particularly for calculated non-radiative relaxation timescales.

### Excited State Population Analysis

After initial excitation in the red edge of the *B* band of the two chlorophyll species, we calculated the population evolution of the *B* (*B*(*t*)), *Q*_*x*_ (*X*(*t*)), and *Q*_*y*_ (*Y*(*t*)) bands as shown in Fig. 5 by carrying out the NA-ESMD simulations. The *B* band in this population analysis is composed of excited states *S*_3_ − *S*_10_, the *Q*_*x*_ band is *S*_2_, and the *Q*_*y*_ band is *S*_1_. Population of each band at a given time was calculated as the average over all the trajectories. We assume a first-order irreversible transfer from *B* → *Q*_*x*_ with a rate constant *k*_1_ and from *Q*_*x*_ → *Q*_*y*_ with a rate constant *k*_2_, yielding the sequential transfer pathway 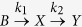.

The population of each excited state can then be given as


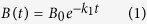



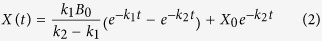



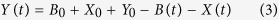


where *B*_0_, *X*_0_, and *Y*_0_ denote the initial (*t* = 0) populations of the *B*, *Q*_*x*_, and *Q*_*y*_ bands, respectively. The fitting procedure is carried out via a least-squares regression analysis. The rate constants and the corresponding time constants (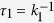, 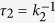) are presented in Table 1. Figure 5 shows the results of fitting the simulations-derived data using these modeled rate constants. A convergence check was performed on the time constants from the population fit, and is presented in the *Methods* section.

From Table 1, a number of interesting similarities and differences between ChlA and ChlB can be noted. The overall time constant for internal conversion from *B* → *Q*_*y*_ is higher for ChlB (267 fs) than for ChlA (227 fs). The NA-ESMD simulations in this work are carried out using Langevin equations with a friction coefficient of *γ* = 2 ps^−1^ without any explicit solvent effects. Moreover, larger gaps between calculated states compared to the respective experimental values, typically assume slower theoretical relaxation rates. Therefore, rather than the absolute rate constants, the relative changes in internal conversion rates for different pathways as well as between different chlorophyll species can be more meaningfully compared with the experimental data. Promising comparisons can thus be drawn in the overall trends in the dynamics. Well in agreement with the theoretical predictions, the measured time constant for the *B* → *Q*_*y*_ relaxation in ChlB (162 fs) is slightly higher than that in ChlA (143 fs). Furthermore, the ratio of the time constants between ChlB and ChlA is also found to be comparable between the simulations (1.18) and the experiments (1.13). We note that in the experimental set-up, ChlB is excited at higher energy states within the *B*-band as compared to ChlA. However, this difference is not expected to exert any drastic influence on the observed comparison between the two species because of the extremely fast intra-*B*-band relaxation. This contention has also been verified for ChlA via both the simulations and the experiments (see Figure S1-S2 from Supplementary Information).

Our results show the calculated time constants for *B* → *Q*_*x*_ to be shorter than those for *Q*_*x*_ → *Q*_*y*_ internal conversion, as listed in Table 1 for both chlorophyll species. The experiments carried out in the present work do not resolve the *B* → *Q*_*x*_ and the *Q*_*x*_ → *Q*_*y*_ pathways separately. However, the timescales of the internal conversion to *Q*_*y*_ from *B* and *Q*_*x*_ in ChlA have been probed in different solvents in an earlier experimental study^17^. It was found that the time-constants for *B* → *Q*_*y*_ internal conversion (*τ*_*tot*_) were 146 fs (ethyl ether), 186 fs (ethyl acetate), 220 fs (tetrahydrofuran), and 260 fs (dimethyl formamide). Additionally, the time constants for *Q*_*x*_ → *Q*_*y*_ internal conversion (*τ*_2_) were 100 fs (ethyl ether), 132 fs (ethyl acetate), 138 fs (tetrahydrofuran), and 226 fs (dimethyl formamide)^17^. Although the *B* → *Q*_*x*_ internal conversion rates could not be directly measured, this pathway could be assumed to exist as the time constants for *B* → *Q*_*y*_ pathway are significantly larger than those for *Q*_*x*_ → *Q*_*y*_ pathway. With this assumption, the time constants for *B* → *Q*_*x*_ internal conversion from their experimental data can be roughly estimated to be 46 fs (ethyl ether), 54 fs (ethyl acetate), 82 fs (tetrahydrofuran), and 34 fs (dimethyl formamide), in good agreement with the trend predicted by NA-ESMD simulations for ChlA. The ratio of *B* → *Q*_*y*_ to *Q*_*x*_ → *Q*_*y*_ time constants in Shi *et al.* is ~1.4–1.6 for the three solvents with similar dielectric constants (ethyl ether, ethyl acetate, and THF), and ~1.15 for dimethyl formamide, which has a much higher dielectric constant^17^. The ratio of calculated *B* → *Q*_*y*_ to *Q*_*x*_ → *Q*_*y*_ time constants in this study is 1.77 for ChlA and 1.28 for ChlB, in general agreement with experiment even though solvent effects are not taken into account in the NA-ESMD simulations. In the work by Reimers *et al.*, timescales of 99–134 fs were estimated for the *Q*_*x*_ → *Q*_*y*_ internal conversion pathway in ChlA^12^, also in good agreement with our ChlA simulations. Although similar behavior is predicted for ChlB, to the best of our knowledge, there is no corresponding experimental data for ChlB from other earlier studies, precluding a direct comparison with our calculated results. Finally, when sequenced into *B* → *Q*_*x*_ and *Q*_*x*_ → *Q*_*y*_ pathways, the corresponding time constants for ChlA and ChlB compare distinctly. In ChlB, the *B* → *Q*_*x*_ pathway has a shorter time constant, and the *Q*_*x*_ → *Q*_*y*_ pathway has a longer time constant ChlA. For a more comprehensive understanding of such an interesting comparison between ChlA and ChlB, as discussed in the next section, we have analyzed the localization of the electronic wavefunction at specific atoms, groups and fragments of each of the molecules.

### Analysis of Spatial Distribution of Photoexcited Wavefunction During Internal Conversion

**Figure 1:**
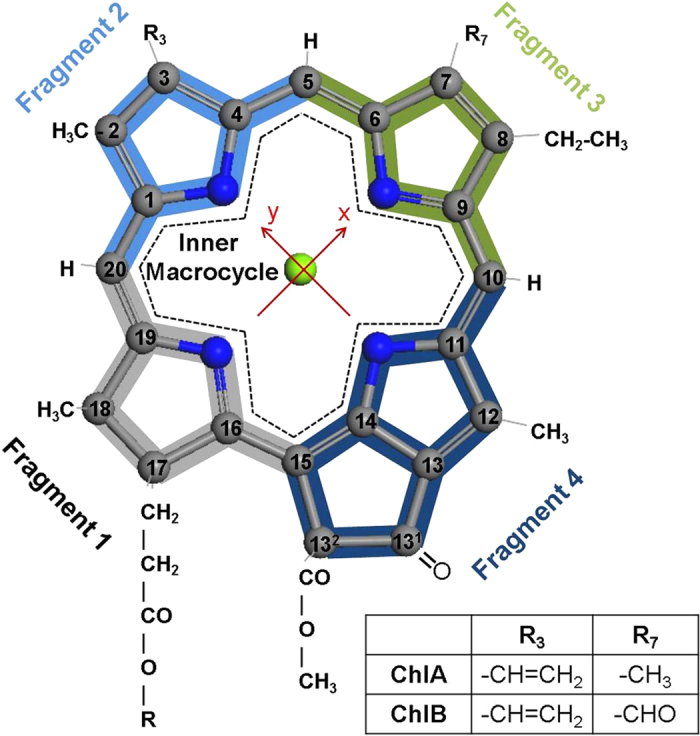
Molecular structure of chlorophylls *a* and *b*. Carbon atoms are labeled according to the IUPAC convention for porphyrins, with the standard x- and y- axes of Gouterman’s four-orbital theory shown. Constituent groups at the *R*_3_ and *R*_7_ positions are shown in the lower right corner of the diagram. Chlorophyll phytyl tails (R) are not shown.

**Figure 2:**
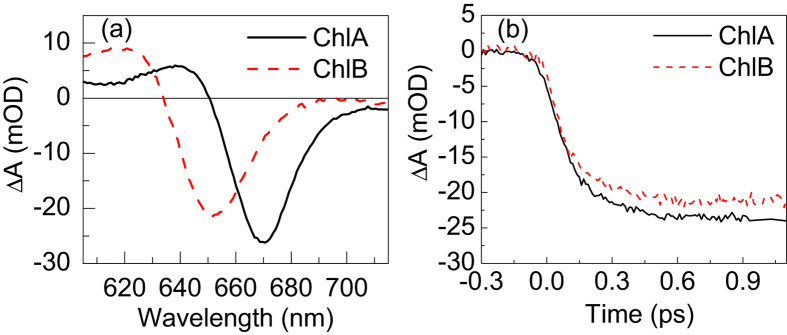
Transient absorption of ChlA (solid black curve) and ChlB (dashed red curve) dissolved in ethanol, probed in the *Q*_*y*_ region after excitation at 442 nm. (**a**) Typical transient absorption spectra recorded at delay time of 1 ps. (**b**) Typical transients recorded at 672 nm and 652 nm for ChlA and ChlB, respectively. The samples were diluted to OD ~ 0.8 at the pump wavelength. The signals corresponding to ChlB are multiplied by two.

**Figure 3:**
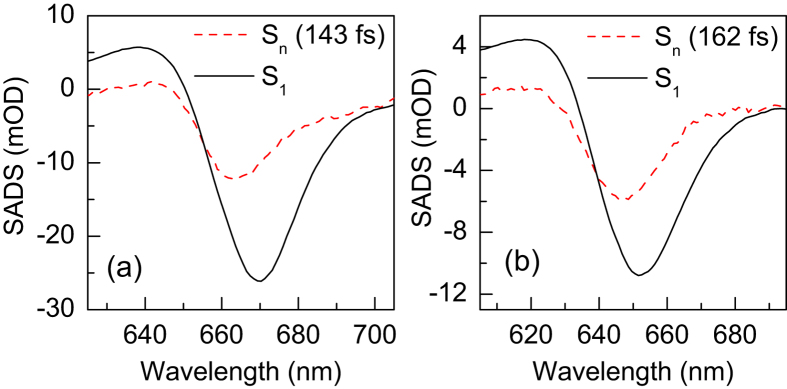
Species-associated absorption difference spectra of the *S*_*n*_ state and *S*_1_ state of Chl resulting from fitting a two-component sequential kinetic model *S*_*n*_ → *S*_1_ to the experimental transient absorption measured in a 2.5 ps window, for (a) ChlA in ethanol and (b) ChlB in ethanol. The *S*_*n*_ → *S*_1_ lifetime is shown in parentheses. The *S*_1_ state is quasi-stationary (fixed 5 ns lifetime).

**Figure 4:**
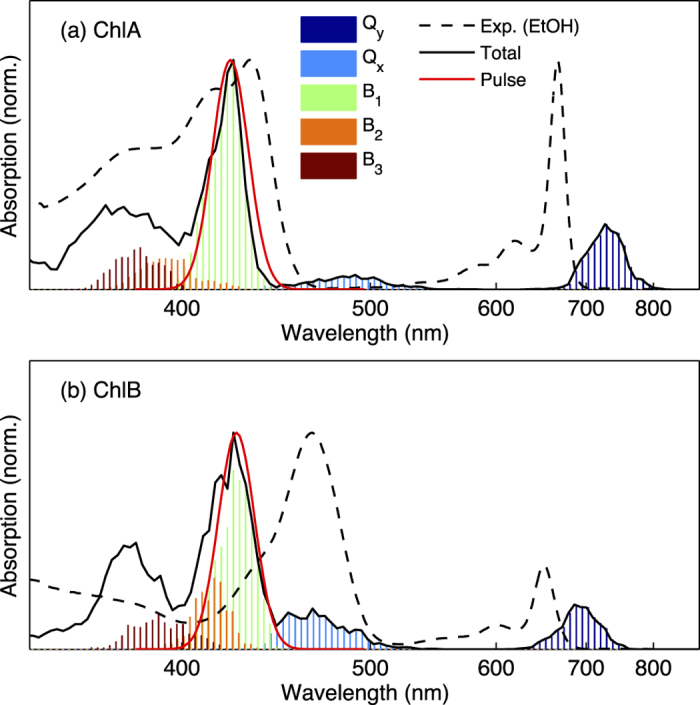
Excited state energy histogram of the ground state structures of (a) ChlA and (b) ChlB. The average of 500 conformations shows relative oscillator strengths of the *Q*_*y*_, *Q*_*x*_, *B*_1_, *B*_2_, and *B*_3_ bands, and total oscillator strength (solid black), compared to experimental spectra in ethanol (dashed black). Within the simulations, each Chl molecule was initially excited at the *B*_1_ band using a Gaussian pulse (red). In the experimental study, both of the Chl molecules were excited at 442 nm.

**Figure 5:**
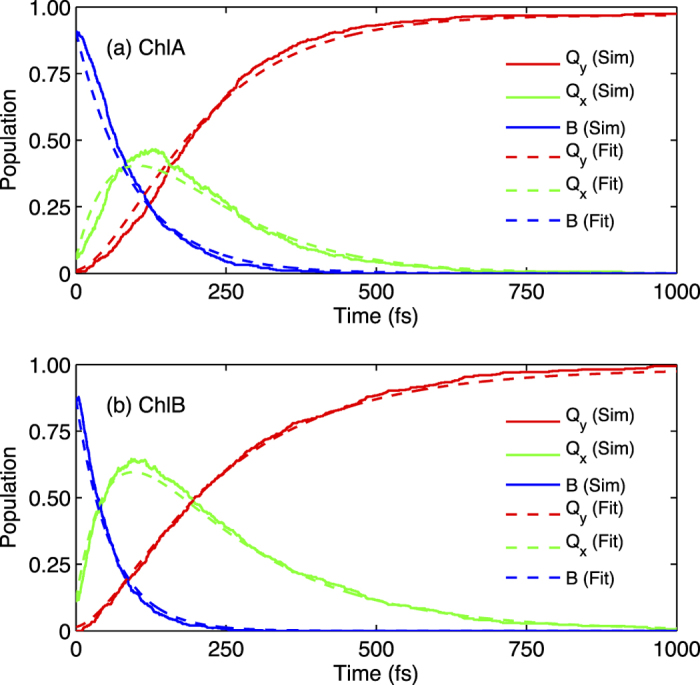
Averaged excited state populations of *Q*_*y*_ (red), *Q*_*x*_ (green), and *B* (blue) bands in (a) ChlA and (b) ChlB species during a 500 trajectory nonadiabatic excited-state molecular dynamics (NA-ESMD) simulation (Sim). Population model fit (first-order, irreversible) is also shown for *Q*_*y*_, *Q*_*x*_, and *B* bands (Fit). The excited state simulations were run for 1 ps, and began with an excitation pulse centered around the *B* band.

**Figure 6:**
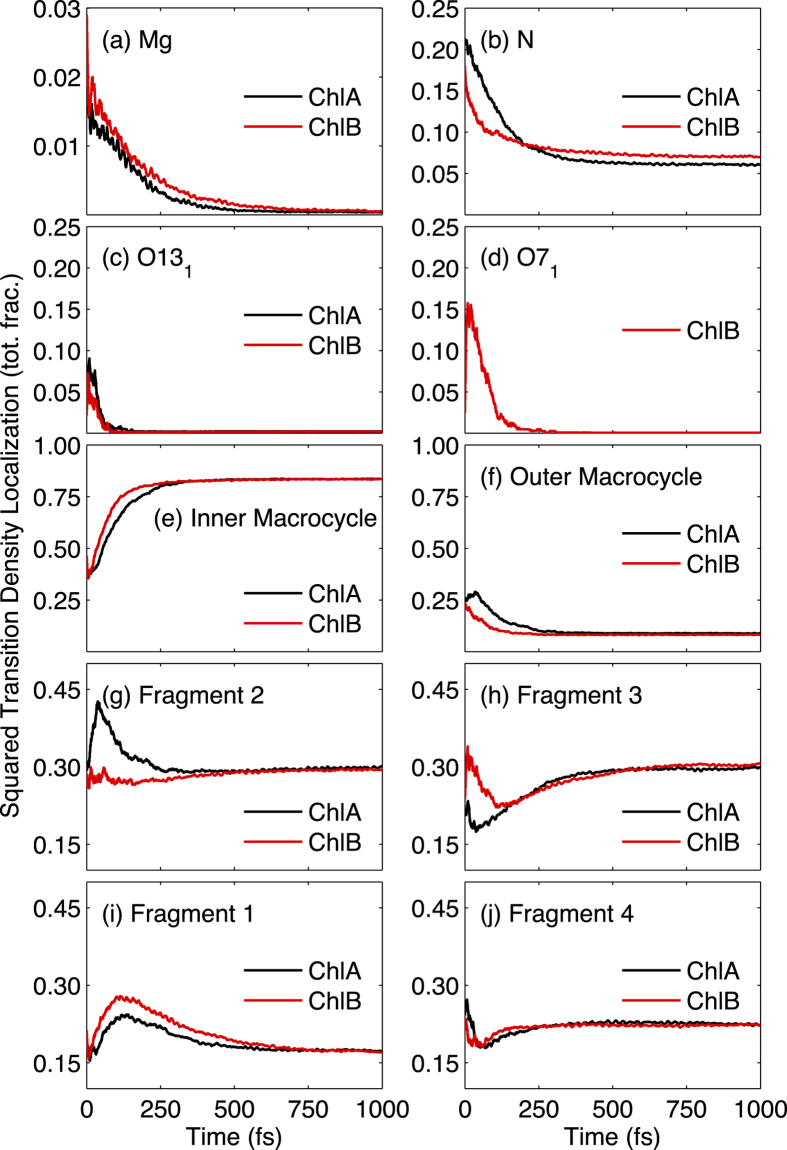
Calculated averaged squared transition density in ChlA (black) and ChlB (red) localized on the (a) central Mg atom, the (b) four N atoms, the (c) O13_1_ atom, the (d) O7_1_ atom, the (e) inner carbon macrocycle, the (f) outer carbon macrocycle, and fragments (i) 1, (g) 2, (h) 3, and (j) 4, during 1 ps NA-ESMD simulations. The outer carbon macrocycle is defined as the total carbon macrocycle minus the inner carbon macrocycle.

The differences between the molecular structures of the two chlorophyll species studied in this work are only in terms of side-groups, as shown in Fig. 1 where compared to ChlA, ChlB has a carbonyl oxygen (O7_1_) in R7 substituent group. Yet they lead to noticeable differences in the absorption spectra and calculated rates of internal conversion. (See Figs 4 and 5 and Table 1.) To determine how these structural motifs influence the excited state dynamics, we analyzed the localization of the excited state wavefunction on specific atoms, functional groups, and regions of the molecule in terms of their time evolution as the system relaxes from the *B* → *Q*_*x*_ → *Q*_*y*_ bands in each of the chlorophyll species. Figure 6(a–d) shows the time evolution of averaged and normalized transition density localized on some important individual atoms for each of the chlorophyll species during the excited-state dynamics. In addition, the summed transition density over certain groups of atoms is depicted in Fig. 6(e,f). The total carbon macrocycle is defined as the carbon atoms comprising the porphyrin ring structure (shown as highlighted atoms in Fig. 1 excluding the N atoms), whereas the “inner macrocycle” (denoted by the dotted line in Fig. 1 excluding the N atoms) consists of atoms C1, C4, C5, C6, C9, C10, C11, C14, C15, C16, C19, and C20, and the “outer macrocycle” is comprised of carbon atoms from the total carbon macrocycle minus those in the inner macrocycle.

We further analyze the delocalization of the excited state wavefunction localization in the molecules as split in four fragments, which are devised according to the previous theoretical and experimental knowledge of chlorophyll excited state structure. The Gouterman four-orbital model for porphyrin-type molecules suggests some basic characteristics of the typical chlorophyll excited states. This model states that the major molecular orbital (MO) configurational contributions are the [2 1 1 0] and [1 2 0 1] singly excited configurations for the *Q*_*y*_ and *B*_*y*_ excited states, and the [1 2 1 0] and [2 1 0 1] singly excited configurations for the *Q*_*x*_ and *B*_*x*_ excited states, with the difference between *Q* and *B* excited states being the relative phase of the configurations. (The ground state configuration here is represented as [2 2 0 0]). The Gouterman model also theorizes that these transitions are polarized along the porphyrin x- and y-axes, as defined in Fig. 1, such that the *Q*_*y*_ and *B*_*y*_ transitions are polarized along the y-axis, and the *Q*_*x*_ and *B*_*x*_ transitions are polarized along the x-axis^10^,^11^. While the Gouterman model is not completely accurate for ChlA and ChlB due to their asymmetry along the porphyrin axes^12^, it is useful in our analysis to split the porphyrin ring into four easily defined fragments. As defined in Fig. 1, we split the porphyrin ring into four arbitrary fragments (Fragments 1–4). Fragments 1 and 3 are in the direction of *Q*_*x*_ whereas the fragments 2 and 4 are in the direction of *Q*_*y*_. The atoms connecting two fragments (C5, C10, C15, and C20) were split equally between them. Constituent side-groups (excluding H-atoms) were also included in the fragments. Thus, using this fragment model, we are able to determine the extent to which the structural differences (O7_1_ in fragment 3 of ChlB) influence the overall excited state wavefunction delocalization.

The time evolution of the averaged transition density localized on some important atoms (Fig. 6(a–d)), groups of atoms (Fig. 6(e,f)) and all the four fragments (Fig. 6(g–j)) allows for a clear comparison between the two chlorophyll species. We first present major similarities and differences between both chlorophyll species from this figure.

In both chlorophyll species, the overall process of *B* → *Q*_*y*_ conversion is associated with the transfer of localized transition density from the N atoms, the carbonyl O atoms, and the outer carbon macrocycle to the inner carbon macrocycle, and the central Mg atoms play a relatively insignificant role in the evolution of the excited state wavefunction. The O7_1_ (ChlB) atom provides a significantly greater impact on localized transition density than the O13_1_ atom, which are found in both chlorophylls. ChlA exhibits stronger changes in the transition density on the fragment 2 oriented along the porphyrin y-axis than that on fragments 1 and 3 lying along the x-axis. In contrast to the characteristics of ChlA, the total transition density in ChlB is found to be relatively undisturbed in fragments 2 and 4 (the porphyrin y-axis), while showing stronger variation in fragments 1 and 3 (the porphyrin x-axis).

We are also interested in associating the time-evolution of the electronic transition density localization with the different electronic excited state-specific features and the energy transfer between them. (See Figure S3-S4 in the Supplementary Information.) We note several main features from this state-specific analysis. First, the *B* bands in both ChlA and ChlB have localized transition density on the N atoms, the O7_1_ (ChlB only) and O13_1_ atoms, and the outer carbon macrocycle. Second, in both ChlA and ChlB, a transition density flux along the x-oriented axis in fragments 1 and 3 is seen during the *Q*_*x*_ → *Q*_*y*_ transfer pathway, and the y-oriented axis in fragments 2 and 4 does not play a significant role.

### Internal Conversion Mechanisms

**Figure 7:**
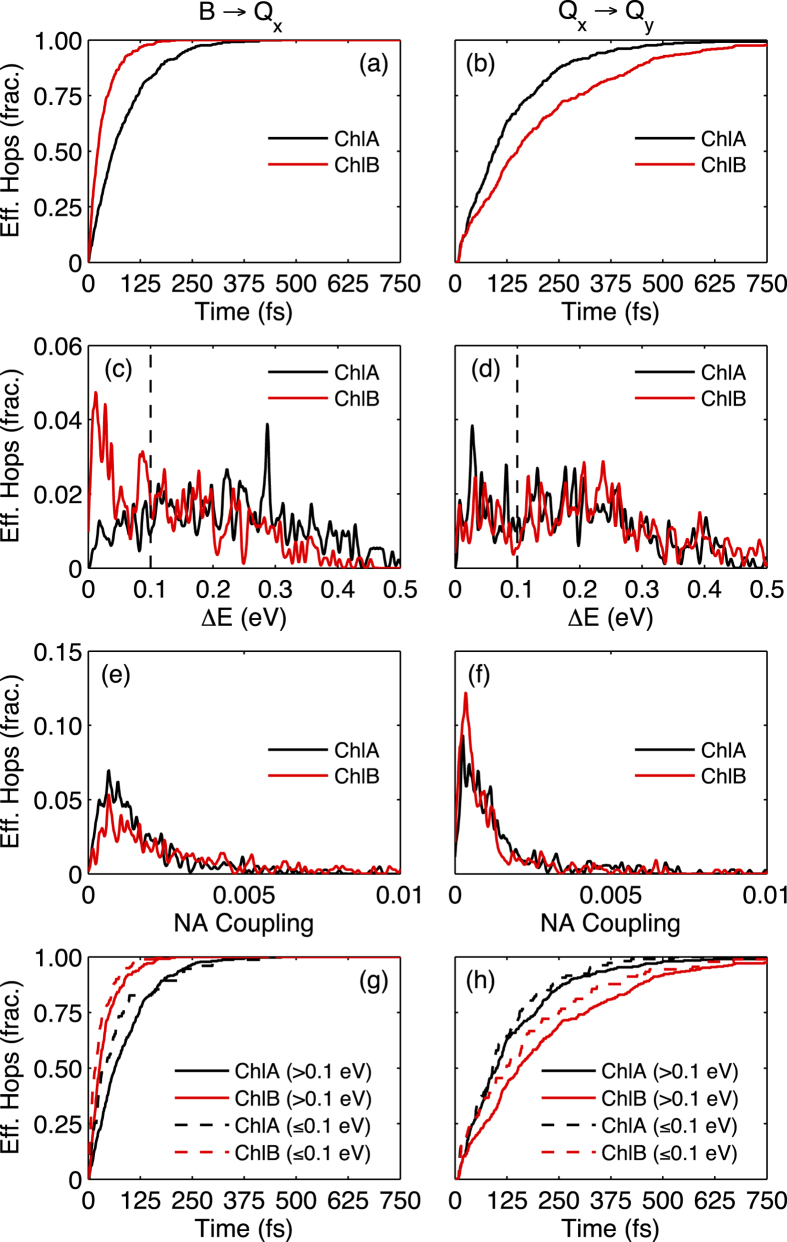
Excited state potential energy surface (PES) events during 1 ps NA-ESMD simulations of ChlA (black) and ChlB (red). Times of effective (**a**) *B* → *Q*_*x*_ and (**b**) *Q*_*x*_ → *Q*_*y*_ hops, energy gaps (Δ*E*) between (**c**) *B* and *Q*_*x*_, and (**d**) *Q*_*x*_ and *Q*_*y*_ excited states during effective hops, frequency of nonadiabatic coupling magnitude during effective (**e**) *B* → *Q*_*x*_ and (**f**) *Q*_*x*_ → *Q*_*y*_ hops, and times of effective hops with Δ*E* > 0.1 eV (solid) and Δ*E* ≤ 0.1 eV (dashed) for (**g**) *B* → *Q*_*x*_ and (**h**) *Q*_*x*_ → *Q*_*y*_. In (**a**,**b**,**g**,**h**), *t* = 0 refers to the moment the donor excited state (*B* – (a) and (g), *Q*_*x*_ – (b) and (h)) is initially populated on each trajectory.

In Fig. 7, various properties of the *B* → *Q*_*x*_ and *Q*_*x*_ → *Q*_*y*_ pathways are plotted, including the distributions of the time (fs) and energy gap, Δ*E* (eV), of effective excited state hops, and a distribution of the nonadiabatic coupling magnitudes at these times. From the cumulative plot in Fig. 7(a), it becomes evident that the effective hops from *B* → *Q*_*x*_ occur on a shorter time-scale in ChlB than those in ChlA. In ChlB, most effective hops have occurred within 200 fs of the initial *B* excitation, while ChlA continue to have effective hops until 500 fs. Also, when comparing Fig. 7(a,b), all *B* → *Q*_*x*_ effective hops occur on a shorter time-scale than *Q*_*x*_ → *Q*_*y*_ effective hops. This observation is consistent with the calculated absorption spectra, which show a much greater overlap of *B* and *Q*_*x*_ bands in ChlB, and is also consistent with the proposed mechanism of sequential internal conversion from *B* → *Q*_*x*_ → *Q*_*y*_. Figure 7(c) further shows that during the effective hops in *B* → *Q*_*x*_ transfer in ChlB, the exciton appears to pass close to the seam of the conical intersection. In contrast, *B* → *Q*_*x*_ transfer evolves further away from the conical intersection seam in ChlA. This *B* → *Q*_*x*_ relaxation pathway through the conical intersection seam can explain the shorter lifetime for ChlB with respect to ChlA. Figure 7(e,f) show the distribution of nonadiabatic (NA) coupling magnitudes for *B*/*Q*_*x*_ and *Q*_*x*_/*Q*_*y*_ excited states during effective hops. The *B*/*Q*_*x*_ NA couplings in Fig. 7(e) are related to the fact that ChlB effective hops occur close to the seam of the conical intersection, thus stronger NA couplings are more common in ChlB.

The properties of *Q*_*x*_ → *Q*_*y*_ pathway are more consistent among the two molecules. Distributions of effective hopping times and energy gaps shown in Fig. 7(b,d) are relatively similar between chlorophyll species, the only exception being the hopping times of ChlB which occur on a longer time-scale. This is most likely due to the greater separation between the *Q*_*x*_ and *Q*_*y*_ absorption peaks in the calculated absorption spectra. In contrast to the *B*/*Q*_*x*_ NA couplings, the *Q*_*x*_/*Q*_*y*_ NA couplings in Fig. 7(f) are weaker, with effective hops occurring further from the conical intersection. We also note that ChlB has weaker overall NA couplings for *Q*_*x*_/*Q*_*y*_ transfer, explaining the slower *Q*_*x*_ → *Q*_*y*_ internal conversion rate compared to ChlA. This finding supports the strong mixing between *Q*_*x*_/*Q*_*y*_ excited states found in ChlA by Reimers *et al.*^12^, since we show that *Q*_*x*_ → *Q*_*y*_ internal conversion in ChlA is faster due to effective hops occurring closer to the conical intersection. For both *B* → *Q*_*x*_ and *Q*_*x*_ → *Q*_*y*_, most of the effective hops occur with an excited state energy gap of 0.0–0.4 eV. Effective hops occured at an average Δ*E* of 0.227 eV and 0.142 eV for *B* → *Q*_*x*_ internal conversion in ChlA and ChlB, respectively, and an average Δ*E* of 0.206 eV and 0.231 eV for *Q*_*x*_ → *Q*_*y*_ internal conversion in ChlA and ChlB, respectively. These are significantly smaller gaps than those initially shown in the absorption spectrum in Fig. 4, whose values are 0.42 eV in ChlA and 0.26 eV in ChlB for Δ*E* between *B*/*Q*_*x*_, and 0.82 eV in ChlA and 0.86 eV in ChlB for Δ*E* between *Q*_*x*_/*Q*_*y*_. Therefore, the nuclear motion on the excited states after photoexcitation takes place in a portion of the phase space not accessible through the ground state dynamics.

We have previously mentioned that our analysis of electronic transitions reveals certain transitions to likely occur close to the conical intersection seam between the corresponding excited states (see Fig. 7(c,d)). In order to further analyze this aspect, we adopt an arbitrary limit of Δ*E* < 0.1 eV to separate the cases of strict conical intersections or their immediate regions and the cases with larger Δ*E* values. With this separation in mind, Fig. 7(g,h) show separately the time-dependence of the accumulated number of *B* → *Q*_*x*_ and *Q*_*x*_ → *Q*_*y*_ transitions for trajectories whose transitions take place at Δ*E* < 0.1 eV and Δ*E* > 0.1 eV. As we observe, energy transfer through conical intersection seams takes place at slightly earlier times than the transitions far away from the conical intersection. That is, the nuclear motions on either the *B* and *Q*_*x*_ states initially lead the molecular system close to the *B*/*Q*_*x*_ and *Q*_*x*_/*Q*_*y*_ conical intersection seams respectively.

The difference in hopping times close to the conical intersection is quantifiable. In ChlA, the *B* → *Q*_*x*_ transition occurs in an average time of 71 fs when Δ*E* < 0.1, and in 86 fs when Δ*E* > 0.1. The *Q*_*x*_ → *Q*_*y*_ transition takes place over 117 fs for Δ*E* < 0.1, and 138 fs for Δ*E* > 0.1. Similar results were found for ChlB, wherein the *B* → *Q*_*x*_ (*Q*_*x*_ → *Q*_*y*_) transition takes place in an average time of 28 fs (172 fs) and 40 fs (215 fs) for Δ*E* < 0.1 and Δ*E* > 0.1, respectively. Next, the molecules move towards regions of the configuration space with larger Δ*E* and consequently less efficiencies in energy relaxation. The percentage of energy transfer occurring through conical intersection regions (Δ*E* < 0.1 eV) and larger Δ*E* values is distinct for ChlA and ChlB. For *B* → *Q*_*x*_ (*Q*_*x*_ → *Q*_*y*_) internal conversion, 17% (26%) of hops in ChlA and 43% (21%) of hops in ChlB occur close to the conical intersection.

## Conclusions

By utilizing the nonadiabatic excited-state molecular dynamics (NA-ESMD) method, which allows for an accurate study of photoinduced dynamics in pigment molecules, we calculated and analyzed the internal conversion rates in the two most important photosynthetic molecules, chlorophyll *a* and chlorophyll *b*. The simulation results predict the overall internal conversion from the high-energy excited states in the *B* band to the lowest excited state *Q*_*y*_ to be slightly faster in ChlA as compared to ChlB. Experimental measurements carried out on ChlA and ChlB with transient absorption pump-probe techniques yield the *B* → *Q*_*y*_ time constants to be 143 fs and 162 fs, respectively, thereby corroborating the theoretical predictions. Time-constants for the *B* → *Q*_*x*_ and *Q*_*x*_ → *Q*_*y*_ transfer processes separately are also found to exhibit a trend in agreement with previous experimental data for ChlA.

In order to determine how structural differences in chlorophyll species contributed to differences in internal conversion rates, we analyzed the localization of the excited state electronic wavefunction on specific atoms and groups in each pigment, as well as four fragments splitting the porphyrin ring along its respective x- and y- axes as defined in Goutermans four-orbital model. From this localized transition density analysis, we see striking differences between the ChlA and ChlB species, due to the extra carbonyl oxygen (O7_1_) along the porphyrin x-axis in ChlB. This analysis of the excited state wavefunction redistribution in the two chlorophyll species results in several observations. First, the overall electronic transition density primarily moves from the “outer carbon macrocycle”, as well as the Mg, N, and carbonyl O atoms to the “inner carbon macrocycle” during the internal conversion from *B* → *Q*_*x*_ → *Q*_*y*_, and second, the extra carbonyl O atom (O7_1_ in ChlB) is strongly influential in directing the localized transition density, as shown in the four fragment transition density models. This carbonyl oxygen thus plays a significant role in the redistribution of the excited state wavefunction, thereby contributing to the differences in internal conversion time constants between ChlB and ChlA. While the internal conversion rates between chlorophyll species are inherently controlled by structural differences, the energy transfer pathways in the two species are distinct, particularly in the *B* → *Q*_*x*_ transfer pathway. ChlB follows a pathway that passes close to the seam of the conical intersection during *B* → *Q*_*x*_ internal conversion, while chlorophyll *a* does not. On the contrary, for *Q*_*x*_ → *Q*_*y*_ internal conversion, both chlorophylls pass far away from the conical intersection. Despite that, ChlA has revealed exploring regions with higher nonadiabatic couplings than ChlB.

**Figure 8:**
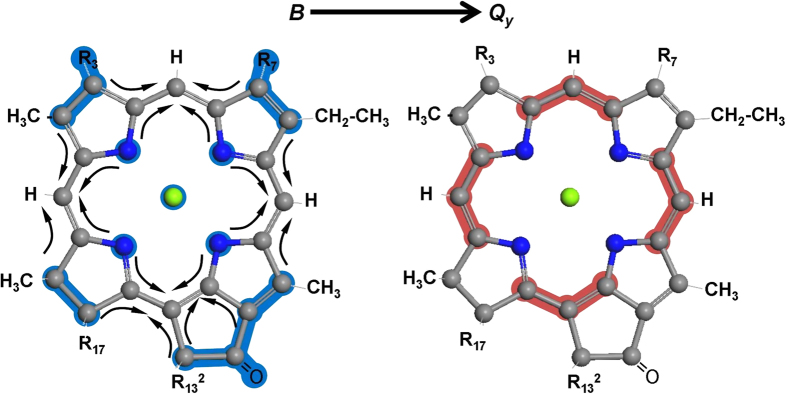
Schematic of predominant redistribution of the transition density localization during the overall *B* to *Q*_*y*_ conversion for both chlorophyll *a* and *b*.

Furthermore, we report differences in the transient intramolecular energy redistributions that can take account of the experimental differences in the *B* → *Q*_*x*_ → *Q*_*y*_ internal conversion rates. The different substituents between chlorophyll species have been shown to play significant roles during the early times of the electronic energy relaxation that involve *B* → *Q*_*x*_ energy transfer. Despite that, the internal conversion process in both the Chls culminates with the exciton localized mainly on the common “inner carbon macrocycle”. Figure 8 depicts a schematic representation of the overall internal conversion process. It exhibits the evolution of time-dependent electronic wavefunction presenting a complex superposition of adiabatic electronic states across 500 trajectories, which cannot be approximated by a simple analysis of adiabatic states at ground state optimal geometry. It may then be inferred that any potential impact that different side-groups of different chlorophyll species can have on the intermolecular energy transfer processes that participate in the photosynthetic process will depend upon the relative time scales between the intermolecular and intramolecular energy transfer processes. If intermolecular energy transfers are concomitant with the intramolecular internal conversion, the transient localization of the exciton on the O7_1_ atoms can modify the intermolecular process according to the chlorophyll species. Otherwise, if the intermolecular energy transfer is subsequent to it, the transfer from an exciton localized on the inner macrocycle seems to be common to the two types of Chls.

Thus, our non-adiabatic dynamics simulations along with experimental ultrafast probes provide extensive information on intramolecular non-radiative relaxation of photoexcited chlorophylls *a* and *b*, which are common to a variety of biological photosynthetic complexes. Even given smaller electron-vibrational couplings compared to common organic conjugated chromophores, these molecules are able to efficiently dissipate about 1 eV of electronic energy into heat on the timescale of around 200 fs. This is achieved via selective participation of specific atomic groups and complex global migration of the wavefunction from the outer to inner ring, which may have important implications for biological light-harvesting function. Further studies concerning the interplay and relative time scales of the inter- and intramolecular energy transfers are in progress.

## Methods

### Experimental Methods

Chlorophyll *a* and *b* was obtained from Sigma Aldrich as dry samples. The samples were dissolved in ethanol and adjusted to an optical density of 0.8 at 440 nm in a 2 mm pathlength cell. Transient absorption (TA) pump-probe measurements were performed using a femtosecond time-resolved spectrometer system employing a Ti:Sapphire regenerative amplifier (Legend, Coherent) seeded by mode-locked pulses from a Ti:Sapphire oscillator (Vitesse, Coherent). Part of the amplified output laser pulse centered at 800 nm (with 0.8 mJ pulse energy at 1 kHz and 150 fs pulse duration) was used to seed a commercial optical parametric amplifier (TOPAS-white, Light Conversion) to generate 442 nm as a result of doubling of 884 nm generated by the OPA. A prism-pair pulse compressor inside the OPA compresses the 442 nm pulses to around 55 fs which have been measured using a home-built autocorrelator and also corroborated by the recorded lifetime of coherent artifact in the pump-probe signal. This 442 nm pulse of 100 nj energy and 10 nm bandwidth was used to excite the Soret band of Chls. A small fraction (1%) of the amplified 800 nm pulse was focused on a 2 mm sapphire window to generate white-light continuum between 442 nm and 790 nm for the probe pulse. The pump and white-light probe pulses were overlapped in the Chl solution in a 2 mm quartz cuvette and the probe beam was focused onto a spectrograph (Horiba Jobin-Yvon) equipped with a CCD array detector (Pixis 100, Princeton Instruments). Absorption difference spectra were recorded at pump-probe delays between −2 and 800 ps with a minimum step of 10 fs. Steady-state absorption spectra were taken before and after the transient absorption measurements to check for the integrity of the sample. All measurements were performed in the dark at room temperature. Kinetic model fitting (global analysis) of the data incorporating convolution with the instrument response and correction for group velocity dispersion and coherent artifact was performed using the program Glotaran 1.3^40^.

### Computational Methods

#### NA-ESMD Background

The NA-ESMD methodology^38^,^41^ provides an accurate and efficient framework to simulate photoinduced dynamics of extended conjugated molecules involving multiple coupled electronic excited states. This is achieved by combining the “fewest switches surface hopping” (FSSH) algorithm^42^,^43^ with “on the fly” analytical calculations of excited-state energies^44^,^45^,^46^, gradients^47^,^48^, and non-adiabatic coupling^38^,^49^,^50^,^51^ terms. All of these quantities are calculated using the collective electron oscillator (CEO) method^52^,^53^,^54^ applied at the AM1 semiempirical level^55^,^56^ in combination with the configuration interaction singles (CIS) formalism to describe correlated excited states^57^. The AM1 semiempirical method and the similar PM3 semiempirical method^56^,^58^ have been used previously to describe electronic and structural properties of porphyrin-based molecules, as well as of chlorins, bacteriochlorins, chlorophylls, and bacteriochlorophylls^59^,^60^,^61^. The AM1 semiempirical method has been shown to correctly reproduce the symmetric environment of the magnesium atom^56^,^62^. Parameters for the central Mg atom in the chlorophylls were chosen based on the force field developed specifically for the bacterial photosynthetic reaction center^62^. Semiempirical methods such as AM1 are chosen for nonadiabatic excited state molecular dynamics calculations due to their computational efficiency. While computational efficiency is improved using semiempirical methods, vertical excitation energies are underestimated using standard semiempirical methods such as AM1^63^. Absolute excitation energies are not important in this study, only relative excitation energies, so these shortcomings are unproblematic. The implementation of the FSSH in the NA-ESMD code has been extensively optimized in order to permit the simulation of nonadiabatic dynamics of molecules consisting of hundreds of atoms on the time scale of tens of picosecond. The nuclei are propagated classically on a potential energy surface which is defined by a single AM1/CIS electronic state at a given time. For this purpose, the Velocity Verlet algorithm combined with a constant-temperature Langevin dynamics algorithm^64^ is used. Electronic transitions from one electronic surface to another are allowed and they are governed by coefficients of the electronic wave function which is meanwhile propagated quantum-mechanically in the adiabatic basis of AM1/CIS states. The variations of the quantum coefficients require a more delicate treatment than the nuclei. This is achieved using a Runge-Kutta-Verner fifth- and sixth-order method^65^. A detailed discussion about the NA-ESMD implementation, advantages, and testing parameters can be found elsewhere^38^,^41^,^66^.

To follow spatial delocalization of the excited state wavefunction across a chromophores atoms and groups, we analyze dynamics of the transition density matrices calculated as


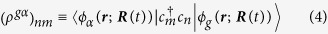


(denoted electronic normal modes) using the ground-state density matrix, where *ϕ*_*g*_(***r***; ***R***(*t*)) and *ϕ*_*α*_(***r***; ***R***(*t*)) are the AM1/CIS adiabatic ground and excited state wave functions, respectively, ***r*** and ***R*** are the electronic and nuclear coordinates, respectively, 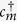
(*c*_*n*_) are creation (annihilation) operators, and *n* and *m* refer to atomic orbital basis functions. Therefore, the diagonal elements (*ρ*^*gα*^)_*nn*_ represent the net change in the distribution of the electronic density induced by optical excitation from the ground state *g* to an electronic excited state *α*^67^. Within the CIS approximation, the usual normalization condition 
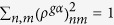
 is fulfilled^46^. In order to obtain the fraction of the transition density localized on different moieties, or arbitrary groups of atoms in the molecule, we can sum up the atomic contributions belonging to each of them as


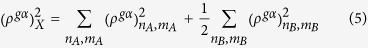


where the index A runs over all atoms localized in the X-moiety, and the index B runs over atoms, if any, which also belong to a second moiety. We have implemented NA-ESMD to simulate the photoexcitation and intramolecular electronic energy redistribution in the two model chlorophylls depicted in Fig. 1. Relevant computational models, schemes and various parameters are described in the next two subsections.

#### Ground State Molecular Dynamics

Prior to the start of the ground-state dynamics, geometry optimization was performed on each chlorophyll molecule (See Fig. 1) including the phytyl tail, at the B3LYP/6-31g(d) level of theory^68^,^69^,^70^ using Gaussian 09^71^. The ground state molecular dynamics simulations were then carried out by running five separate 110 ps long Born-Oppenheimer trajectories at 300 K with a time step Δ*t* = 0.5 fs. The system was heated and allowed to equilibrate to the final temperature of 300 K during the first 10 ps. To describe the motion of the classically treated nuclei, the Langevin equation at constant temperature was utilized with a friction coefficient of *γ* = 2.0 ps^−1^ 64. The friction coefficient is chosen based on numerical testing where *γ* = 2.0 ps^−1^ provided balanced temperature coupling without overdamping the nuclear relaxation during dynamics^66^. In the production run following equilibration, we collected a set of initial positions and momenta for the subsequent excited-state molecular dynamics simulations. Thus, after the initial 10 ps equilibration period, configurations were sampled every 1 ps yielding an ensemble of 500 configurations per chlorophyll species. Both ground and excited state molecular dynamics simulations were performed at the AM1/CIS level of theory.

#### Non-Adiabatic Excited State Molecular Dynamics

Each of the ground-state configurations from the ensemble obtained previously is subjected to an initial photo-excitation by a suitably chosen Gaussian-shaped laser pulse. The initial excited state population is assigned according to a Frank-Condon window defined as *g*_*α*_(***r***; ***R***) = exp[−*T*^2^(*E*_*laser*_ − Ω_*α*_)^2^] where *E*_*laser*_ is expressed in the units of fs^−1^, and Ω_*α*_ represents the energy of the laser centred at the maximum wavelength of the Soret band in the theoretical absorption spectrum (Fig. 4). The laser shape is assumed to be a Gaussian *f*(*t*) = exp(−*t*^2^/2*T*^2^), where the full width at half maximum (FWHM) is given as *FWHM* = Γ ≈ 2.355*T*. Thus, the initial excited state is selected according to the relative values of the *g*_*α*_(***r***; ***R***) weighted by the oscillator strengths of each state *α*.

The peak energy and the FWHM of the laser pulses were chosen as follows: *E*_*ChlA*_ = 2.94 eV and Γ_*ChlA*_ = 0.14 eV for chlorophyll *a*, and *E*_*ChlB*_ = 2.92 eV and Γ_*ChlB*_ = 0.14 eV for chlorophyll *b*. The peak of the laser excitation pulse coincides with that of the lowest excited state in the Soret band (*S*_3_) in each chlorophyll’s spectral histogram. A total of 500 trajectories are propagated independently for 1 ps at 300 K, using a classical time-step of Δ*t* = 0.1 fs and *N*_*q*_ = 4 quantum time steps per classical step to simultaneously propagate the electronic coefficients. During the excited state dynamics, the 10 lowest excited states were propagated for each trajectory. The existence of trivial unavoided crossings has been considered by tracking the identities of states^72^. Convergence of internal conversion timescales has been previously shown to require a minimum of ~400 independent trajectories for adequate statistical averaging to achieve ~5% relative error^66^. A convergence check was also performed for this NA-ESMD data, using unique combinations of batches (each batch contains 100 trajectories) taken randomly from the total pool of 500 excited state trajectories. These sets of data include: 100 trajectories (5 combinations of 1 batch), 200 trajectories (10 combinations of 2 batches), 300 trajectories (10 combinations of 3 batches), 400 trajectories (5 combinations of 4 batches), and 500 trajectories (1 combination of 5 batches). This data from this convergence analysis is presented in Table 2, where the error bars represent the standard deviation. Thus, all results converge to less than 5% relative error with 300 independent trajectories, and less than 3% relative error with 400 independent trajectories.

## Additional Information

**How to cite this article**: Bricker, W. P. *et al.* Non-radiative relaxation of photoexcited chlorophylls: theoretical and experimental study. *Sci. Rep.* **5**, 13625; doi: 10.1038/srep13625 (2015).

## Supplementary Material

Supplementary Information

## Figures and Tables

**Table 1:** Comparison of internal conversion rates obtained from a first-order irreversible population model for *B* → *Q*_*x*_, *Q*_*x*_ → *Q*_*y*_, and *B* → *Q*_*y*_ pathways.

Mol.	*B* ⟶ *Q*_*x*_		*Q*_*x*_ ⟶ *Q*_*y*_		*B* ⟶ *Q*_*y*_
	*k*_1_ (fs^−1^)	*τ*_1_ (fs)	*k*_2_ (fs^−1^)	*τ*_2_ (fs)	*τ*_*tot*_ (fs)
ChlA	0.0101	99.2 ± 1.1	0.00781	128 ± 2	227 ± 3
ChlB	0.0173	57.9 ± 1.4	0.00481	208 ± 3	267 ± 4

Populations of specific excited states were averaged from 500 trajectories in the NA-ESMD simulations. Error bars represent the standard deviation from a convergence check on unique combinations of the NA-ESMD excited states trajectories.

**Table 2:** Comparison of average internal conversion time constants obtained from a first-order irreversible population model for *B* → *Q*_*x*_ (*τ*_1_) and *Q*_*x*_ → *Q*_*y*_ (*τ*_2_) pathways using various numbers of independent trajectories taken randomly from the total pool of 500 NA-ESMD trajectories.

Mol.	Num. ofTraj.	*τ*_1_ (fs)	*τ*_2_ (fs)
ChlA	100	99.3 ± 4.5	128 ± 8
	200	99.2 ± 2.6	128 ± 5
	300	99.2 ± 1.7	128 ± 3
	400	99.2 ± 1.1	128 ± 2
	500	99.2	128
ChlA	100	57.9 ± 5.7	208 ± 12
	200	57.9 ± 3.3	208 ± 7
	300	57.9 ± 2.2	208 ± 5
	400	57.9 ± 1.4	208 ± 3
	500	57.9	208

The error bars represent the standard deviation.
