# Supplementary material for: Non-radiative relaxation of photoexcited chlorophylls: theoretical and experimental study
Source: Sci Rep. 2015 Sep 8;5:13625. doi: 10.1038/srep13625 (PMC4561917; doi:10.1038/srep13625)
Supplement: Supplementary Information [file srep13625-s1.pdf]

Non-radiative relaxation of photoexcited chlorophylls: theoretical and experimental study

William P. Bricker,[a,♯] Prathamesh M Shenai,[b,♯] Avishek Ghosh,[c] Zhengtang Liu,[c] Miriam Grace M. Enriquez,[c] Petar H. Lambrev,[c,d] Howe-Siang Tan,[c] Cynthia S. Lo,[a] Sergei Tretiak,[e] Sebastian Fernandez-Alberti[f]* and Yang Zhao[b] †

[a] *Department of Energy, Environmental and Chemical Engineering, Washington University, Saint Louis, Missouri 63130, USA*

[♯] *These authors contributed equally to this work.*

[b] *Division of Materials Science, Nanyang Technological University, Singapore 639798*

[c] *Division of Chemistry and Biological Chemistry, School of Physical and Mathematical Sciences, Nanyang Technological University, Singapore 637371*

[d] *Biological Research Center, Hungarian Academy of Sciences, 6726 Szeged, Temesvari krt. 62, Hungary*

[e] *Theoretical Division, Center for Nonlinear Studies (CNLS), and Center for Integrated Nanotechnologies (CINT), Los Alamos National Laboratory, Los Alamos, New Mexico 87545, USA*

[f] *Universidad Nacional de Quilmes, Roque Saenz Pea 352, B1876BXD Bernal, Argentina*

Internal Conversion in ChlA Following Excitation of High-Energy B Band States

NA-ESMD Simulations

In addition to the NA-ESMD calculations presented in the main text, we have simulated the internal conversion processes following an initial excitation (366 nm / 3.38 eV) centered on high-energy states within the B band ($S_6 - S_8$) of ChlA. All the other conditions and simulation parameters are identical to those described in the manuscript for ChlA. The high-energy initial excited states are found to exhibit ultrafast relaxation to the lowest excited state ($S3$) within the B band, over a time scale of tens of femtoseconds. For simplicity, we show in Figure S1 only the the rise of the population of the Q_y state under two initial excitation conditions. It can be easily observed that despite the different initial excitations within the B band, Q_y population curves reveal no appreciable differences. We thus assert that the time constants for the $B \rightarrow Q_y$ internal process are relatively similar if the excitation energy resides within the B band.

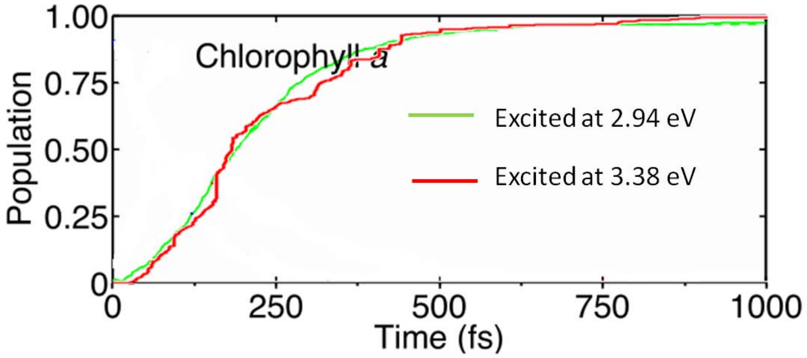

Figure S1: Averaged excited state populations of Q_y bands in chlorophyll a after an initial excitation centered at 2.94 eV (green curve) and 3.38 eV (red curve).

* Electronic address: sfalberti@gmail.com

† Electronic address: YZhao@ntu.edu.sg; Phone: +(65) 65137990

Experimental Set-up and Results

The sample was dissolved in ethanol and adjusted to an optical density of 0.6 in a 1 mm pathlength at 400 nm. Transient absorption pump-probe measurements were recorded using a femtosecond time-resolved spectrometer system employing a Ti:Sapphire regenerative amplifier (Legend Elite, Coherent) seeded by mode-locked pulses from a Ti:Sapphire oscillator (Micra, Coherent). The output laser pulse centered at 800 nm (with 0.8 mJ pulse energy at 1 kHz and 40 fs pulse duration) is frequency doubled using a Type-I BBO crystal to produce 400 nm pump pulse. A small fraction of the 800 nm beam was focused to a 2 mm sapphire window to generate a white light continuum used as probe pulse. The pump and probe beams were overlapped at the sample at 54.7° magic angle polarization. A spectrometer (Acton SP2300, Princeton Instruments) equipped with CCD detector (Pixis 100B, Princeton Instruments) was used to detect the probe signal. The sample was circulated in a 1 mm flow cell to ensure that the pump beam excites different portions of the sample during data collection. Pump pulse energy was attenuated to 20 nJ to avoid photo-degradation of the sample. Transient absorption spectra were collected up to 5000 fs delays withincrements of 10 fs step size. Steady-state absorption spectra of ChlA were taken before and after transient absorption measurement to check for the integrity of the sample. All measurements were performed in the dark at room temperature. Kinetic model fitting (global analysis) of the data incorporating convolution with the instrument response and correction for spectral dispersion was performed using Glotaran 1.3.

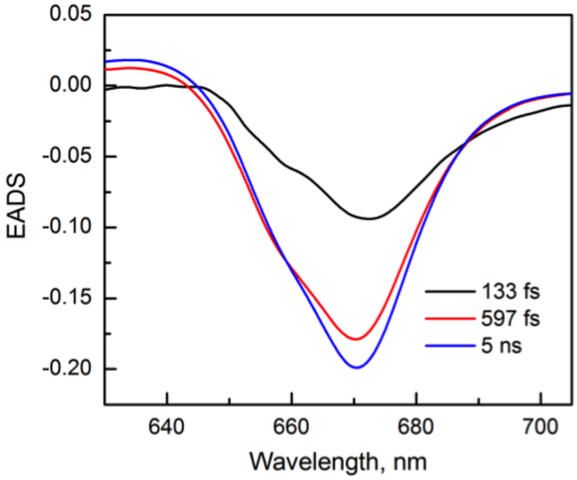

Figure S2: Evolution-associated absorption-difference (EADS) spectra of ChlA at 133 fs, 597 fs and 5 ns

Global fitting analysis based on a sequential irreversible deactivation pathway of excited states was carried out on the transient absorption datasets of ChlA excited at the B band. Three kinetic decay components with lifetimes of 133 fs, 597 fs and a long lifetime (fixed to 5 ns) provided the best fit to the transient data as shown in Figure S2. The linear spectral parameters of the model (the evolution-associated absorption-difference spectra (EADS)) reveal the spectral evolution from one decay component to the next. The EADS are dominated by a negative maximum at 670 nm due to ground state bleaching (GSB) and stimulated emission (SE) of the lowest-energy Q_y transition of ChlA ($S_0 \rightarrow S_1$). The pump-probe spectrum of the vibrationally relaxed S_1 state is expected to have equal negative amplitude contribution from GSB and SE. A secondary feature is the small positive absorption difference away at the edges of the spectral window that signifies excited-state absorption (ESA) from S_1. The first EADS represents the state immediately after pump excitation, which decays with a time constant of 133 fs. The spectrum has smaller amplitude at the negative maximum, due to the lack of SE from the higher excited states, and does not show positive ESA, in contrast with the second and third EADS. Therefore, the 133 fs component is attributed to the overall IC process from the B (S_n) to the Q_y (S_1) band of ChlA. The IC from B (S_n) to Q_x (S_2) was not resolved in the experiment. The second EADS (597 fs) shows both GSB and SE, as well as ESA below 650 nm,but has a slightly smaller amplitude than the final EADS. The 597 fs decay component is thus attributed to relaxation of vibrationally non-equilibrated S_1 excited state. The final component represents decay of the relaxed S_1 state to the ground state. The S_1 lifetime is much longer than the measurement time window and was fixed at 5 ns. In conclusion, the transient absorption measurements on ChlA in ethanol revealed that the IC from $S_n \rightarrow S_1$ state occurs with a time constant of 133 fs, which is comparable to the time constant obtained when the initial excitation at in the red-edge of the B

band.

Specific Excited State Features of the Electronic Transition Density Localization of ChlA and ChlB

In this section, we present the transition density localization split into different excited states for both ChlA (Figure S3) and ChlB (Figure S4). The time-evolution of transition densities localizations on different chosen moieties are depicted separately according to the current excited state (B (S_3 only), Q_x (S_2), and Q_y (S_1)). Here, excited states $S_4 - S_{10}$ are omitted from the B band since the primary pathway to Q_x is from S_3 only. Figure S3 depicts this analysis for ChlA. We notice the erratic nature of the B band compared to the Q_x and Q_y bands, due to the ultrafast depletion of the electronic population of this state after photoexcitation. The B-band population nearly vanishes at about $t = 450$ fs, and is responsible for the transition density localized on the N atoms (although Q_x plays a role here as well), the O13_1 atom, the vinyl group at C3_1, and the outer carbon macrocycle. The initial rise in the B-band's contribution of C3_1, and the outer carbon macrocycle as well as the total transition density in Figure S3 (d) and (f) is due to excited state population transfer from $S_4 \rightarrow S_3$.

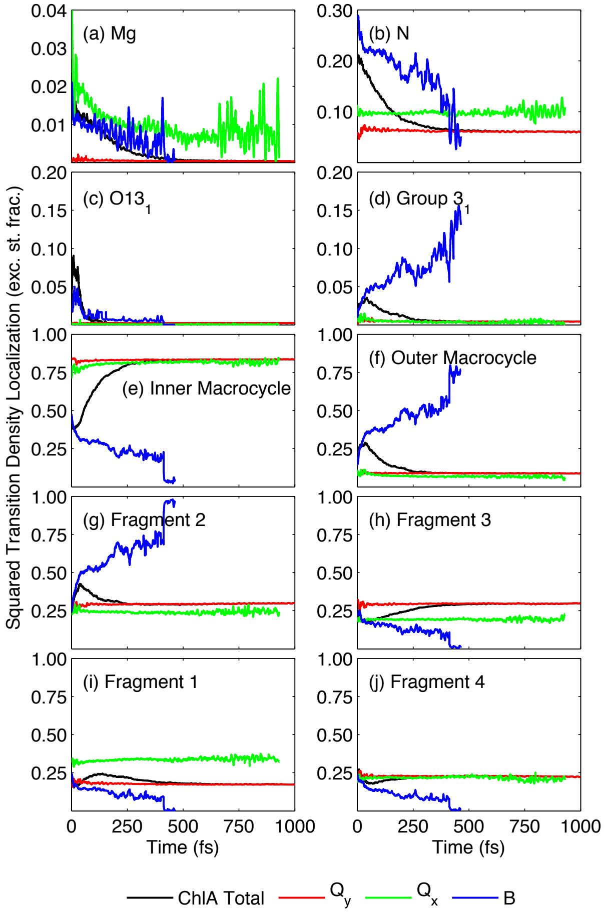

Figure S3: Calculated averaged squared transition density in ChlA (black) localized on the (a) central Mg atom, the (b) four N atoms, the (c) O13_1 atom, the (d) Group 3_1 atoms, the (e) inner carbon macrocycle, the (f) outer carbon macrocycle, and fragments (i) 1, (g) 2, (h) 3, and (j) 4, during 1 ps NA-ESMD simulations. Localized transition density is split between the Q_y (red), Q_x (green), and B (blue) transitions. The outer carbon macrocycle is defined as the total carbon macrocycle - the inner carbon macrocycle.

In Figure S3 (g), the initial increase in the total transition density on fragment 2 is due to the B state contribution localized on the C3_1 vinyl group and outer macrocycle carbon atoms (C2 and C3). On the other hand, B character decays on the rest of the fragments. Significant differences in the localization of the transition density corresponding to Q_x and Q_y bands are found to be mainly in the x-oriented fragments 1 and 3, indicating a transition density flux

from fragment 1 to fragment 3 during the shift in population from $Q_x \to Q_y$. The $Q_x \to Q_y$ transition also involves a decrease in the transition density localization on the N atoms, as seen in Figure S3 (b).

The localized transition density of ChlB's excited states is shown in Figure S4, and the erratic nature of the B band compared to the Q_x and Q_y bands is evident again, even more so than in ChlA. The B band population reduces to zero at nearly $t = 300$ fs. Again, the initial rise in the B band in Figure S4 (d) and (f) is due to excited state population shifting from $S_4 \to S_3$. The B band trends here are similar to ChlA, in that it is responsible for the transition density localized initially on the N atoms, on the O13$_1$ atom, and the outer carbon macrocycle. Besides, Figure S4 (d) shows that the transition density of the B state presents a significant localization on the O7$_1$ atom. The faster $B \to Q_x$ transition respect to ChlA is reflected in faster initial decrease of the transition density localization in all these moieties.

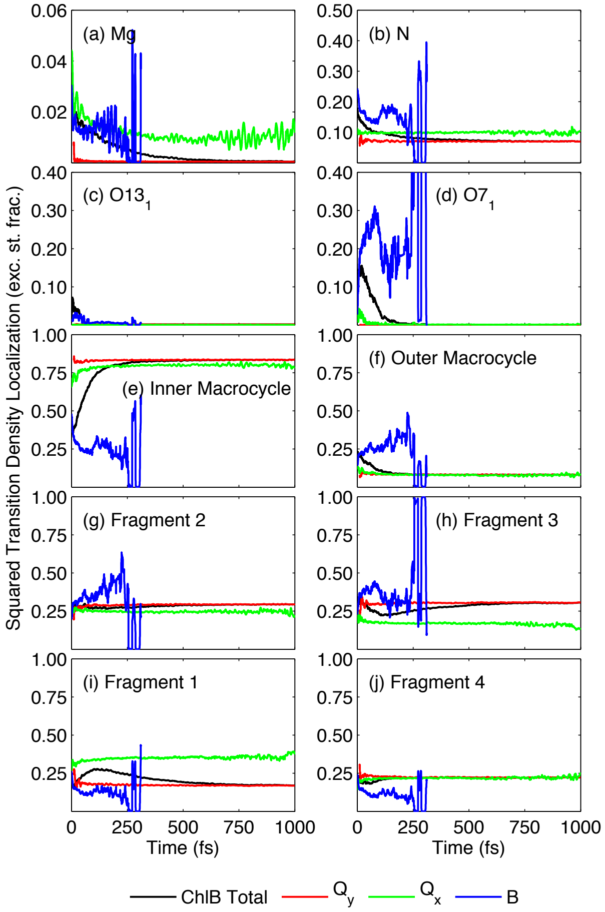

Figure S4: Calculated averaged squared transition density in ChlB (black) localized on the (a) central Mg atom, the (b) four N atoms, the (c) O13$_1$ atom, the (d) O7$_1$ atom, the (e) inner carbon macrocycle, the (f) outer carbon macrocycle, and fragments (i) 1, (g) 2, (h) 3, and (j) 4, during 1 ps NA-ESMD simulations. Localized transition density is split between the Q_y (red), Q_x (green), and B (blue) transitions. The outer carbon macrocycle is defined as the total carbon macrocycle - the inner carbon macrocycle.

As in the case of ChlA, Figure S4 (g) – (j), indicate that fragments 2 and 4 do not show any significant differences in localized transition density between the Q_x and Q_y excited states in ChlB. As it has been also pointed out for ChlA, a significant transition density flux from fragment 1 to fragment 3 is noticed during the shift in population from $Q_x \to Q_y$, being larger in this case than in ChlA. Again, the $Q_x \to Q_y$ transfer pathway also involves a decrease in the transition density localization on the N atoms, as seen in Figure S4 (b), but the magnitude of this effect is less than that found for ChlA.
